# Performance of Two Novel Obesity Indicators for the Management of Metabolic Syndrome in Young Adults

**DOI:** 10.3389/fendo.2021.719416

**Published:** 2021-07-26

**Authors:** Xiaoli Liu, Chunpeng Ma, Fuzai Yin, Rui Wang, Qiang Lu, Na Lu, Chunming Ma

**Affiliations:** ^1^Department of Endocrinology, The First Hospital of Qinhuangdao, Qinhuangdao, China; ^2^Department of Cardiology, The First Hospital of Qinhuangdao, Qinhuangdao, China

**Keywords:** visceral adiposity index, lipid accumulation product, metabolic syndrome, young adults, cross-sectional study

## Abstract

**Background:**

Metabolic syndrome (MetS) is a pathophysiological change based on the abnormal metabolism of many substances. The study aims to investigate the performance of visceral adiposity index (VAI) and lipid accumulation product (LAP) of MetS in young adults.

**Methods:**

448 young adults aged between 19 and 24 years old in Qinhuangdao had been included in this cross-sectional study. Receiver operating characteristic (ROC) curve analyses were used to assess the accuracy of these two obesity indicators for MetS.

**Results:**

The prevalence of MetS was 2.0%. In male subjects, LAP had the highest area under the ROC curve (AUC) value (AUC = 0.963), followed by VAI (AUC = 0.937). In female subjects, LAP also had the highest AUC value (AUC = 0.931), followed by VAI (AUC = 0.861). No significant difference was found between the two obesity indicators (*P* > 0.05).

**Conclusion:**

The two obesity indicators were valuable for the screening of MetS in young adults, and LAP was the simpler of the two.

## Introduction

Metabolic syndrome (MetS) is a pathophysiological change based on the abnormal metabolism of many substances. It has many risk factors related to atherosclerosis, which eventually leads to the occurrence and development of cardiovascular and cerebrovascular diseases.

The early intervention of MetS is important for reducing the risk of developing related diseases. The VAI and LAP are two novel indexes for MetS. VAI and LAP are associated with many chronic diseases. VAI was negatively correlated with insulin sensitivity (1), and was identified as a powerful indicator of prediabetes or diabetes (2). It was positively correlated with the risk of hyperuricemia and nonalcoholic fatty liver disease (3, 4). VAI can predict the incidence of hypertension in prehypertension or healthy people (5). The VAI can be used as a reliable independent risk factor marker for Sexual Dysfunction (ED) as a predictor of visceral adipose dysfunction, and the presence of MeTS has emerged as a risk factor for patients with ED with high VAI levels (6, 7).

Recently, several studies have supported the use of these indexes for the screening of MetS in the healthy population (8–11), the elderly (12, 13), people with polycystic ovary syndrome (14–16) and those with adult growth hormone deficiency (17). The aim of our study was to evaluate the performance of two obesity indicators, VAI and LAP, for identifying MetS in young adults.

## Methods

### Subjects

This is a cross-sectional, population-based study. The participants of this study were patients with metabolic syndrome who came to our hospital from May 18th, 2018. A total of 448 young adults of Han ethnicity, aged between 19 and 24 years old (males/females 235/213), participated in the study. Participants in this study were included in a consecutive manner. The exclusion criteria included the following: 1) patients with other endocrinopathy, such as Cushing’s syndrome and hyperthyroidism; 2) subjects who were taking medication which affect lipid metabolism, such as glucocorticoids and fibrates; 3) subjects with acute and chronic inflammation. This study was approved by the ethics committee of the First Hospital of Qinhuangdao and all subjects had provided written informed consent before the study began.

### Measurements

Anthropometric measurements included height, weight, waist circumference (WC) and Body mass index (BMI). Blood samples were collected after a 10-hour overnight fast. Fasting plasma glucose (FPG) concentration and serum lipid levels were measured.

VAI was calculated as follows (18): male subjects: VAI = (WC/(39.68 + (1.88×BMI)) ×(TG/1.03)×(1.31/HDL); female subjects: VAI = (WC/(36.58 + (1.89×BMI))×(TG/0.81)×(1.52/HDL). LAP was calculated as (WC− 65) × TG for men and (WC− 58) × TG for women (19).

### Definition of Metabolic Syndrome

Participants had to meet three or more of the following five factors: 1) WC ≥ 102 cm (male) and 88 cm (female), 2) FPG ≥ 5.6 mmol/L or had been diagnosed with diabetes, 3) blood pressure ≥ 130/85 mmHg or had been diagnosed with hypertension, 4) TG ≥ 1.7 mmol/L, 5) HDL-C < 1.0 mmol/L (male) and 1.3 cm (female) (20).

### Statistical Analyses

SPSS 11.5 (SPSS 11.5 for Windows; SPSS, Inc., Chicago, IL) was used for data analysis. Continuous variables were expressed as mean ± standard deviation and comparisons between groups was conducted by *t*-test. Kolmogorov Smirnov (KS) test was used to determine the type of data distribution. Comparison prevalence data was performed by*χ* ^2^ analysis. ROC curves were used to show how well they could separate subjects into groups with or without MetS. A test with an area under the curve (AUC) of ≥0.85 was considered accurate (21). The sensitivity and specificity of each obesity indicator was calculated at all possible cut-off points to find the optimal cut-off values. The optimal sensitivity and specificity were the values yielding maximum sums from the ROC curves. *P* < 0.05 was considered statistically significant.

## Results

2.0% of participants were characterised by MetS, male and female shared the same prevalence of MetS (*P >*0.05) (**Table 1**). The age, FPG, TG and LAP between male and female subjects were similar (*P* > 0.05). The levels of BMI, WC, SBP (systolic blood pressure) and DBP (diastolic blood pressure) were all significantly higher in male subjects (*P* < 0.05), while the levels of HDL-C and VAI were significantly lower in male subjects (*P* < 0.05). VAI showed a strong positive correlation with WC, DBP and TG and a negative correlation with HDL-C (*P* < 0.05). LAP showed a strong positive correlation with WC, SBP, DBP and TG and a negative correlation with HDL-C (*P* < 0.05) (**Table 2**).

**Table 1:** General characteristics of participants.

Variables	Males	Females	*t*	*P*
	(n=235)	(n=213)	
Age (year)	19.3 ± 0.8	19.3 ± 0.8	0.131	0.896
BMI (kg/m^2^)	24.6 ± 4.3	23.6 ± 4.1	2.381	0.018
WC (cm)	78.9 ± 11.8	70.1 ± 9.6	8.720	0.000
SBP (mmHg)	116.1 ± 10.6	106.6 ± 10.1	9.688	0.000
DBP (mmHg)	77.2 ± 9.1	71.5 ± 8.0	6.930	0.000
FPG (mmol/L)	4.35 ± 0.45	4.30 ± 0.40	1.271	0.204
TG (mmol/L)	0.74 ± 0.39	0.74 ± 0.26	0.003	0.998
HDL-C (mmol/L)	1.31 ± 0.21	1.46 ± 0.23	6.785	0.000
VAI	0.70 ± 0.59	0.86 ± 0.41	3.166	0.002
LAP	11.4 ± 14.3	9.6 ± 9.2	1.597	0.111

BMI, body mass index; WC, waist circumference; SBP, systolic blood pressure; DBP, diastolic blood pressure; FPG, fasting plasma glucose; TG, triglyceride; HDL-C, high-density lipoprotein cholesterol; VAI, visceral adiposity index; LAP, lipid accumulation product.

**Table 2:** Relationship between VAI, LAP and metabolic syndrome.

Variables	VAI		LAP	
	*r*	*P*	*r*	*P*
WC	0.257	0.000	0.753	0.000
SBP	0.089	0.060	0.277	0.000
DBP	0.104	0.027	0.278	0.000
FPG	0.044	0.357	0.092	0.051
TG	0.932	0.000	0.716	0.000
HDL-C	-0.448	0.000	-0.347	0.000

VAI, visceral adiposity index; LAP, lipid accumulation product; WC, waist circumference; SBP, systolic blood pressure; DBP, diastolic blood pressure; FPG, fasting plasma glucose; TG, triglyceride; HDL-C, high-density lipoprotein cholesterol.

In the male subjects, LAP had the highest AUC value (AUC = 0.963), followed by VAI (AUC = 0.937). LAP had a Youden’s index of 0.85, with an optimal cut-off of 20.1. VAI had a Youden’s index of 0.79, with an optimal cut-off of 1.96 (**Table 3**). In the female subjects, LAP had the highest AUC value (AUC = 0.931), followed by VAI (AUC = 0.861). LAP had a Youden’s index of 0.75, with an optimal cut-off of 13.7. VAI had a Youden’s index of 0.64, with an optimal cut-off of 1.34 (**Table 3**).

**Table 3:** The outcomes of AUC between males and females.

Variables	Cut-off	Sensitivity	Specificity	Youden’s index	AUC (95%CI)	*P*	P*
Males							
VAI	1.96	80.0	99.5	0.79	0.937(0.826~1.000)	0.001	
LAP	20.1	100.0	85.2	0.85	0.963(0.912~1.000)	0.000	0.678
Females							
VAI	1.34	75.0	89.4	0.64	0.861(0.689~1.000)	0.013	
LAP	13.7	100.0	75.5	0.75	0.931(0.829~1.000)	0.003	0.493

AUC, area under curve; CI, confidence interval; VAI, visceral adiposity index; LAP, lipid accumulation product. *Compared with VAI.

## Discussion

The outcomes of this study showed that the performance of LAP and VAI were similar, LAP may be more valuable for MetS in young adults.

Recently, MetS is considered as central obesity. BMI and WC are the main manifestations of MetS, but the sensitivity and specificity of diagnosis of MetS are insufficient (22). VAI is an important index reflecting the distribution of body fat, and the content of visceral fat is closely related to Mets (23). VAI has a certain effect in the diagnosis of MetS (24). The evaluation of visceral adipose tissue has limited availability due to expensive cost and radiation exposure. As the most accurate surrogate marker of visceral adiposity, WC may not be appropriate for identifying MetS (25, 26). However, LAP, as an accurate index for identifying adults at cardiovascular risk (19), is an index based on a combination of WC and TG. In our study, the results showed that LAP was closely related to the components of MetS. These results emphasise the clinical importance of assessing LAP in young adults to identify subjects at high cardiovascular risk.

VAI is an index estimated by anthropometricand metabolic parameters. Among elderly, VAI showed the lowest discriminatory power for MetS (12). Among Chinese rural adults, the VAI AUC for the screening of MetS was less than that of LAP in both men and women (27). However, VAI is not superior to LAP in our study which was consistent with previous results.

### Limitations

On the one hand, LAP was derived from studies of white, non-Hispanic black and Mexican American subjects, but VAI was derived from Caucasian populations (18, 19). Their suitability for other populations needs to be further investigated. On the other hand, the incidence rate of MetS in this study was low, which may be related to the limited sample size.

In summary, two novel obesity indicators were demonstrated to be effective indicators for the screening of MetS in young adults, with LAP being the simpler of the two.

## Data Availability Statement

The original contributions presented in the study are included in the article/supplementary material. Further inquiries can be directed to the corresponding author.

## Ethics Statement

The studies involving human participants were reviewed and approved by The First Hospital of Qinhuangdao. The patients/participants provided their written informed consent to participate in this study.

## Author Contributions 

XL, CPM conceived of the study, and FY and RW participated in its design and coordination and QL, NL and CMM helped to draft the manuscript. All authors contributed to the article and approved the submitted version.

## Conflict of Interest

The authors declare that the research was conducted in the absence of any commercial or financial relationships that could be construed as a potential conflict of interest.
